# Orthopaedic clinics: Spine

**Published:** 2008

**Authors:** Sudhir Kumar

**Affiliations:** Head, Department of Orthopaedics, University College of Medical Sciences & GTB Hospital, New Delhi - 110 095, India. E-mail: profsudhirkumar@gmail.com

What I like about this book is that it is a good tool to learn about the examination of the spine for the residents and it serves as a quick reference on his way to examining a patient with spinal disease and disorder. The book is easy to read and each chapter is well elaborated with abundant illustrations, figures and drawings. It also has a chapter on “How to infer examination findings,” which gives a comprehensive method to diagnose a particular injury or disease of the spine, which may have evolved over a period of time. There is an added advantage of a video format, which facilitates learning for the medical students and the residents. In my opinion, the book is useful and of great help to the residents in strengthening their concept in clinical methods and improving the quality of case presentation. I recommend this book for the medical students and the trainees at all levels and also to the general practitioners who treat patients with spinal problems and may then refer these patients to an orthopaedic surgeon.

